# Molecular Evolution of Protein Sequences and Codon Usage in Monkeypox Viruses

**DOI:** 10.1093/gpbjnl/qzad003

**Published:** 2023-12-12

**Authors:** Ke-Jia Shan, Changcheng Wu, Xiaolu Tang, Roujian Lu, Yaling Hu, Wenjie Tan, Jian Lu

**Affiliations:** State Key Laboratory of Protein and Plant Gene Research, Center for Bioinformatics, School of Life Sciences, Peking University, Beijing 100871, China; Sinovac Biotech Ltd., Beijing 100085, China; NHC Key Laboratory of Biosafety, National Institute for Viral Disease Control and Prevention, Chinese Center for Disease Control and Prevention, Beijing 100052, China; State Key Laboratory of Protein and Plant Gene Research, Center for Bioinformatics, School of Life Sciences, Peking University, Beijing 100871, China; NHC Key Laboratory of Biosafety, National Institute for Viral Disease Control and Prevention, Chinese Center for Disease Control and Prevention, Beijing 100052, China; Sinovac Biotech Ltd., Beijing 100085, China; NHC Key Laboratory of Biosafety, National Institute for Viral Disease Control and Prevention, Chinese Center for Disease Control and Prevention, Beijing 100052, China; State Key Laboratory of Protein and Plant Gene Research, Center for Bioinformatics, School of Life Sciences, Peking University, Beijing 100871, China

**Keywords:** Mpox virus, Positive selection, *OPG027*, Accelerated evolution, Codon usage bias

## Abstract

The monkeypox virus (mpox virus, MPXV) epidemic in 2022 has posed a significant public health risk. Yet, the evolutionary principles of MPXV remain largely unknown. Here, we examined the evolutionary patterns of protein sequences and codon usage in MPXV. We first demonstrated the signal of positive selection in *OPG027*, specifically in the Clade I lineage of MPXV. Subsequently, we discovered accelerated protein sequence evolution over time in the variants responsible for the 2022 outbreak. Furthermore, we showed strong epistasis between amino acid substitutions located in different genes. The codon adaptation index (CAI) analysis revealed that MPXV genes tended to use more non-preferred codons compared to human genes, and the CAI decreased over time and diverged between clades, with Clade I > IIa and IIb-A > IIb-B. While the decrease in fatality rate among the three groups aligned with the CAI pattern, it remains unclear whether this correlation was coincidental or if the deoptimization of codon usage in MPXV led to a reduction in fatality rates. This study sheds new light on the mechanisms that govern the evolution of MPXV in human populations.

## Introduction

The monkeypox virus (mpox virus, MPXV) epidemic in 2022 has caused substantial public health risks. MPXV is a linear double-stranded DNA virus that belongs to the Poxviridae family, Chordopoxvirinae subfamily, and *Orthopoxvirus* genus [1]. The genome of MPXV is approximately 197 kb in length and encodes around 200 genes [2]. MPXV can infect various animal species, including humans, non-human primates, and rodents [3–6]. Similar to variola virus (VARV) and vaccinia virus (VACV) in the *Orthopoxvirus* genus, MPXV can lead to human disease and death.

MPXV was initially discovered in a Danish animal facility in 1958 [6], and it was first isolated from a human case in the Democratic Republic of the Congo in 1970 [7]. Prior to 2022, MPXV was predominantly endemic in Central and Western African countries, with sporadically reported instances in other regions resulting from importations [8–13]. The first human case of the 2022 MPXV outbreak was reported in the United Kingdom on May 7th, 2022 [14]. The global outbreak of MPXV was declared an international public health emergency on July 23rd, 2022. According to the World Health Organization, as of September 11th, 2023, a total of 90,439 confirmed cases from 115 countries and regions had been reported during the 2022–2023 outbreak.

**Figure 1:**
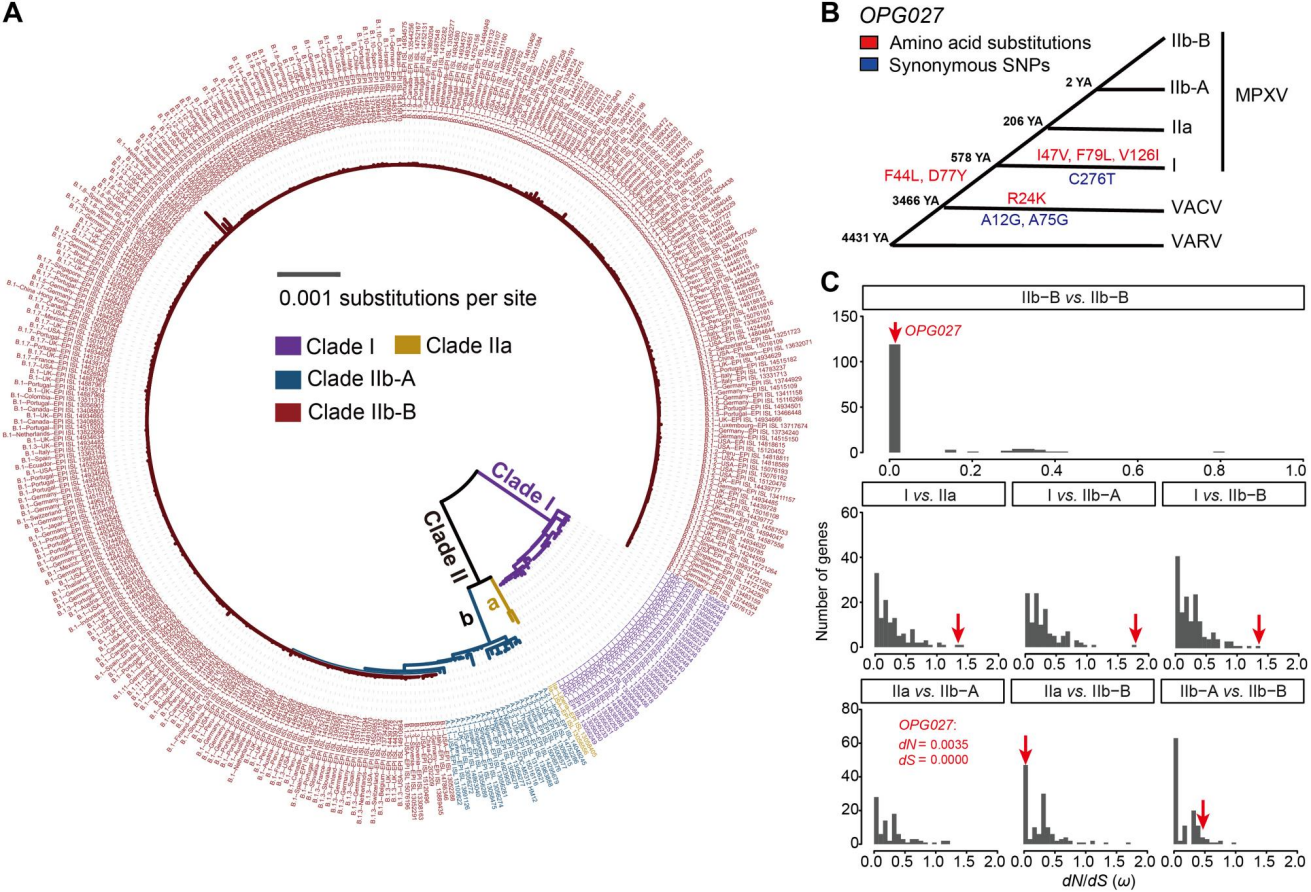
Positive selection on *OPG027* during the evolution of MPXV **A**. Phylogenetic tree of MPXV strains from different clades. The phylogenetic tree was constructed using the maximum likelihood method based on whole-genome alignments of MPXV. **B**. The amino acid substitutions (in red) and synonymous SNPs (in blue) of *OPG027* in the evolutionary process of MPXV. The divergent time of Clades IIb-A and IIb-B was retrieved from Nextstrain (https://nextstrain.org/), and the other divergent time was obtained from data reported by Babkin and his colleagues [59]. **C**. The distribution of the median *dN*/*dS* ratio (*ω*) between two sequences of each gene from different MPXV clades. The red arrow indicates the *ω* value of *OPG027*. It is noteworthy that only one nonsynonymous mutation was observed in the comparisons between IIa and IIb-A strains, with a *dS* value of 0 and a *dN* value of 0.0035 for *OPG027*. YA, years ago; *dN*, nonsynonymous substitutions per nonsynonymous site; *dS*, synonymous substitutions per synonymous site; SNP, single nucleotide polymorphism; MPXV, monkeypox virus; VARV, variola virus; VACV, vaccinia virus.

Based on phylogenetic analysis, MPXV is classified into two major clades: Clade I (also known as the “Central African” clade) and Clade II (also known as the “West African” clade) [15]. Clade II is further subdivided into the IIa and IIb subclades (**Figure 1**A and B). The MPXV variants collected in the 2017–2018 outbreak correspond to subclade IIa or lineage A in subclade IIb (IIb-A) [16,17]. The majority of the MPXV variants responsible for the 2022 outbreak belong to subclade IIb-B, which are phylogenetically more closely related to the variants transported from Nigeria to the United Kingdom, Israel, and Singapore in 2018–2019 than to the variants collected during the 2017–2018 Nigeria outbreak [14,15]. However, sporadic cases of IIb-A.2 sublineage have also been reported in the 2022 outbreak [14,15].

The mutation rate of orthopoxviruses is typically 1–2 substitutions per genome per year [18]. Nevertheless, the evolutionary analysis has revealed about 50 nt differences in the 2022 variants compared with the 2018–2019 variants, which is 6–12 times greater than expected based on the typical orthopoxvirus mutation rate [14]. Moreover, an excess of TC>TT and GA>AA mutations has been identified between the two clades of variants. The increased substitution rate observed in the 2022 MPXV genomes is hypothesized to result from genome editing by apolipoprotein B messenger RNA (mRNA) editing catalytic polypeptide-like 3 (APOBEC3) enzymes, which cause C>T mutations if editing occurs in the sense strand and G>A mutations if editing occurs in the antisense strand [14,15,19]. Considering that the *APOBEC3* gene family is present not only in primates but also in various non-primate species, including many rodents and other small mammals [20,21], it is worth noting that these animals are deemed probable natural hosts for MPXV [22]. As a result, the excessive TC>TT and GA>AA mutations observed in the 2022 MPXV variants could have emerged due to APOBEC3 editing triggered by cryptic circulations within human populations or non-human animals [14].

Despite these recent discoveries, our understanding of the evolutionary principles of MPXV remains limited. In this study, we first identified signatures of positive selection on MPXV genes. Subsequently, we investigated epistasis in the 2022 outbreak-causing MPXV variants. Finally, we examined MPXV codon usage patterns and their potential impact on fatality rates. Our findings reveal accelerated evolution in the MPXV variants causing the 2022 outbreak and prompt caution regarding the potential relationship between deoptimized codon usage and the observed decrease in fatality rates throughout the evolutionary process of MPXV.

## Results

### Positive selection on *OPG027* in Clade I of MPXV

To detect the signatures of positive selection, we downloaded 2789 MPXV genome sequences from the National Center for Biotechnology Information (NCBI) [23] and Global Initiative on Sharing All Influenza Data (GISAID; https://www.gisaid.org, as of November 13th, 2022) [24]. We categorized these sequences into four clades (I, IIa, IIb-A, and IIb-B) using Nextclade [25]. For each gene, we conducted pairwise comparisons between clades (a total of six pairs of clades) by calculating the *dN* (nonsynonymous substitutions per nonsynonymous site), *dS* (synonymous substitutions per synonymous site), and *dN*/*dS* (*ω*) values across all pairwise comparisons between sequences from two different clades. Most comparisons resulted in *ω* < 1, indicating that purifying selection is the predominant force driving MPXV gene evolution (Figure 1C). Nevertheless, we detected 12 genes with median *ω* > 1 in at least one pairwise comparison between genomes from two distinct clades (**Table 1**). Notably, four of these genes (*OPG002*, *OPG023*, *OPG027*, and *OPG031*) are associated with anti-host immunity. Moreover, *OPG027* exhibited *ω* > 1 in comparisons between Clade I and all three lineages of Clade II (IIa, IIb-A, and IIb-B). However, this gene displayed *ω* < 1 in all pairwise comparisons between lineages within Clade II (IIa, IIb-A, and IIb-B) when *dS* > 0. These data suggest that *OPG027* was subject to positive selection during the divergence of Clades I and II, while undergoing purifying selection during the differentiation among lineages within Clade II.

**Table 1:** Genes with median *ω* > 1 in pairwise comparisons between genomes from different clades of MPXV

Gene	Protein	Clade pair	Median ***ω*** (2.5% and 97.5% quantiles)
***OPG002***	CrmB secreted TNF-alpha-receptor-like protein	IIa *vs.* IIb-B	1.07 (0.00, 2.51)
*OPG003*	Ankyrin repeat protein (25)	I *vs.* IIa	1.01 (0.41, 2.05)
***OPG023***	Ankyrin repeat protein (2)	I *vs.* IIa	1.09 (0.84, 1.45)
***OPG027***	Host range protein and type 1 interferon inhibitor	I *vs.* IIa	1.32 (0.90, 1.73)
		I *vs.* IIb-A	1.77 (1.35, 2.18)
		I *vs.* IIb-B	1.32 (0.43, 2.06)
***OPG031***	Interleukin-1 receptor antagonist	I *vs.* IIa	1.34 (1.00, 1.63)
*OPG056*	Extracellular-enveloped virus maturation protein	IIa *vs.* IIb-A	1.16 (1.00, 1.33)
		IIa *vs.* IIb-B	1.33 (0.44, 2.00)
*OPG074*	Intracellular enveloped virion morphogenesis protein	IIa *vs.* IIb-A	1.11 (0.37, 1.48)
*OPG118*	Early transcription factor 70 kDa subunit	IIa *vs.* IIb-B	1.68 (1.67, 1.68)
*OPG145*	DNA helicase	IIa *vs.* IIb-A	1.05 (0.52, 1.27)
		IIa *vs.* IIb-B	1.05 (0.52, 1.41)
*OPG189*	Ankyrin repeat protein (25)	I *vs.* IIb-A	1.11 (0.41, 1.39)
*OPG198*	Ser/Thr kinase	I *vs.* IIb-B	1.17 (0.44, 1.47)
*OPG205*	Ankyrin repeat protein (44)	IIa *vs.* IIb-A	1.19 (0.89, 1.70)

*Note*: The four genes in bold (*OPG002*, *OPG023*, *OPG027*, and *OPG031*) are putatively associated with anti-host immunity. MPXV, monkeypox virus; CrmB, cytokine response modifier B; TNF, tumor necrosis factor.

*OPG027* in MPXV (orthologous to *C7L* in VACV), plays a critical role in determining host range and inhibiting host antiviral activity [23,26–28]. C7L in VACV can target human sterile alpha motif domain containing 9 (SAMD9) proteins, an evolutionarily conserved antiviral factor, to combat host restriction [29]. *OPG027* (*C7L*) is highly conserved in mammalian poxviruses [26], with only six amino acid differences in a sequence of 150 amino acids between OPG027 in MPXV and C7L in VACV (Figure S1). To better understand the different selective forces acting on *OPG027* between Clades I and II, we analyzed the variants in *OPG027* across the 1952 complete and high-quality MPXV genomes, using VACV and VARV as outgroups (Figure 1B; Table S1). We polarized the mutations using the parsimonious method and only focused on mutations that are fixed or nearly fixed (frequency > 90%) within a clade. The branch from the last common ancestor of Clades I and II to Clade II showed no such substitutions. However, the branch leading to Clade I displayed three nonsynonymous substitutions (I47V, F79L, V126I) and one synonymous change (C276T) that had high frequencies (Figure 1B; Table S1). Furthermore, we employed CODEML [30] to identify positive selection signals in *OPG027* (see Materials and methods). By fitting the M8a (neutral and negative selection) and M8 (neutral, negative selection, and positive selection) models, we observed that the M8 model (lnL = −633.729, np = 52) significantly outperformed the M8a model (lnL = −635.826, np = 51) (likelihood ratio test, *P* = 0.04), indicating positive selection on this gene.

We predicted the structure of OPG027 in MPXV based on the structure of C7L in VACV [29] and found that two variant sites (I47V and V126I) were located in the *β*-sheets (Figure S1). Interestingly, the F79 site, part of the hydrophobic loop in C7L, is crucial for viral growth in human cells and is likely directly involved in SAMD9 binding [29]. Although the F79 site is evolutionarily conserved [29], the F79L substitution was observed in Clade I of MPXV (Figure S1), potentially driven by an evolutionary arms race related to viral replication or immune evasion. However, further research is needed to understand the implications of these amino acid changes and any potential epistatic effects between these substitutions.

### Accelerated protein evolution in 2022 outbreak-causing MPXV variants

To decipher the evolutionary trends of the current circulating MPXV variants, we analyzed 756 IIb-B genomes with precise collection dates throughout the 2022 outbreak. The median number of substitutions in an MPXV genome relative to the reference genome (NCBI: NC_063383, collected in August 2018 in Rivers State, Nigeria) was 69, with a range of 67 to 81 at the 2.5th and 97.5th percentiles, respectively. Similarly, the median numbers of synonymous and nonsynonymous single nucleotide polymorphisms (SNPs) were 27 and 33, with 95% confidence intervals (CIs) of 27–30 and 32–41, respectively. TreeTime [31] analysis of these 756 MPXV genomes yielded a genomic-scale average substitution rate of (6.17 ± 0.86) × 10^−5^ substitutions/site/year.

**Figure 2:**
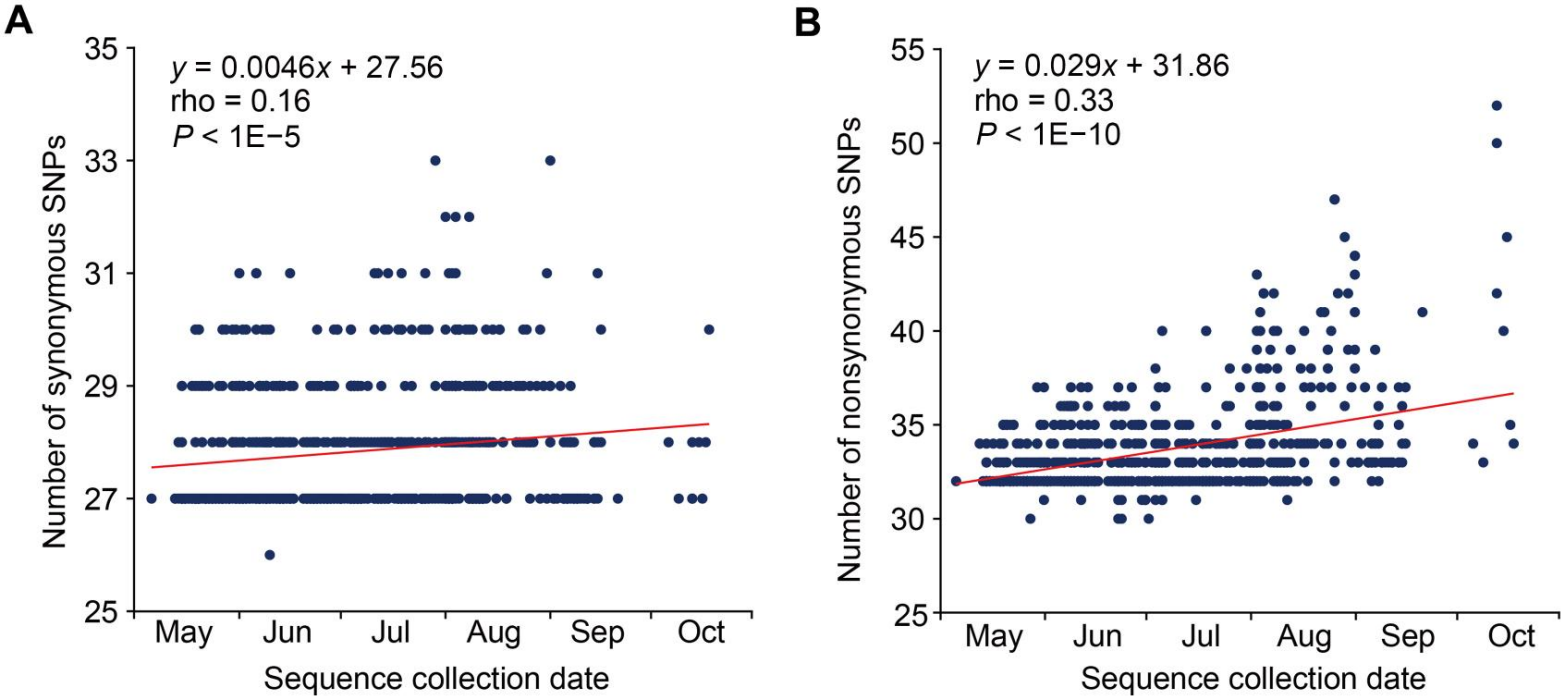
Accumulation of synonymous and nonsynonymous substitutions over time in Clade IIb-B genomes during the 2022 outbreak A total of 756 MPXV genomes with precise collection dates were analyzed. The Y-axis represents the number of synonymous (**A**) or nonsynonymous (**B**) substitutions in a strain collected on a specific day relative to the reference genome (NCBI: NC_063383). Spearman’s correlation coefficient (rho) between the number of substitutions in a strain and time is presented. Additionally, linear regression analyses were performed for the number of substitutions in a strain over time (with May 7th, 2022 assumed as day 1).

As expected, the number of substitutions in an MPXV genome increased over time from May 7th, 2022, when the initial human case of the 2022 MPXV outbreak was identified (**Figure 2**A and B). Notably, within the 2022 outbreak MPXV strains, nonsynonymous substitutions exhibited a stronger correlation with time compared with synonymous substitutions (Spearman’s rho: 0.33 *vs.* 0.16, *P* = 0.0008, Fisher’s method). Additionally, the linear regression slope for nonsynonymous substitutions and time (assuming May 7th, 2022 as day 1) was significantly steeper than that of synonymous substitutions (slope: 0.029 *vs.* 0.0046, *P* = 0.001). These patterns persisted even when we calculated the median number of substitutions for strains collected on each day and conducted the correlation analysis [rho: 0.55 *vs.* 0.34 (nonsynonymous *vs.* synonymous), *P* = 0.037; slope: 0.037 *vs.* 0.0057 (nonsynonymous *vs.* synonymous), *P* = 0.001]. These findings support the notion that the 2022 outbreak-causing MPXV variants have experienced accelerated protein sequence evolution.

### Epistasis in 2022 outbreak-causing MPXV variants

**Figure 3:**
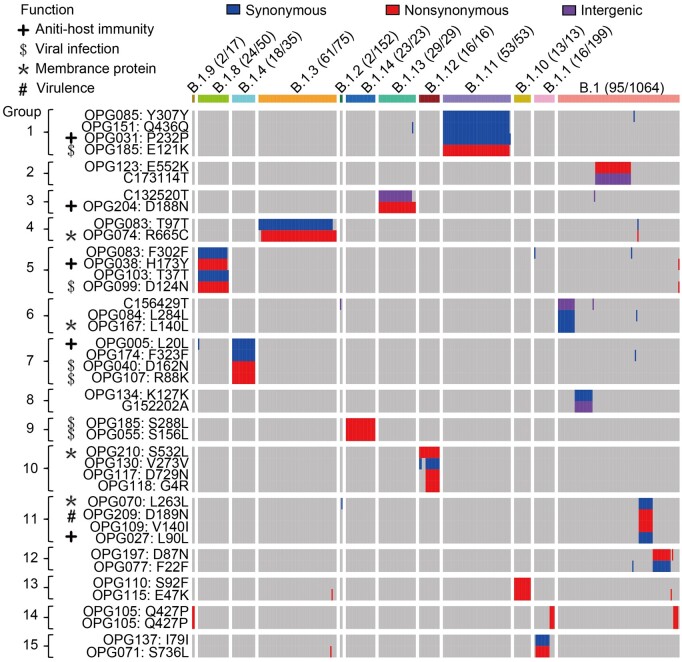
LD for the 15 linked SNP groups (*r*^2^ ≥ 0.8) found in Clade IIb-B of MPXV The Y-axis illustrates the intergenic SNPs or amino acid changes resulting from SNPs within a given gene. Both the A82405C and A82406T mutations resulted in the Q427P change (CAA>CCT) in OPG105. Colored blocks represent SNPs, while gray areas indicate no mutation at that position within a lineage. The number of genome sequences containing the SNPs demonstrating linkage is provided for each lineage, divided by the total number of genome sequences within that lineage (these numbers are presented in parentheses). In total, data from 1726 genomes in Clade IIb-B are presented. LD, linkage disequilibrium.

A salient observation for genetic variants of severe acute respiratory syndrome coronavirus 2 (SARS-CoV-2) is the strong linkage among many mutations within these genomes [32–34]. We questioned whether such a pattern also existed in MPXV variant strains, and to explore this, we examined the linkage disequilibrium (LD) patterns among MPXV SNPs (Figure S2). Specifically, we focused on SNPs with frequencies ranging from 0.005 to 0.8 in the 1873 MPXV genomes of Clade IIb-B, and we only included SNP pairs with *r*^2^ ≥ 0.8 in the LD analysis. These criteria led to the identification of 41 substitutions (comprising 16 synonymous, 21 nonsynonymous, and 4 intergenic ones) that together formed 15 linkage groups. These groups were distributed across 11 lineages within Clade IIb-B. Of the 21 nonsynonymous mutations, 11 were situated in genes associated with the viral infection, virulence, membrane protein, or anti-host immunity (**Figure 3**; Table S2).

Out of the 15 linkage groups, 13 contained at least one nonsynonymous mutation, and six were composed of at least two tightly linked nonsynonymous substitutions located in different genes (Figure 3; Table S2). For instance, we observed a strong linkage between H173Y in OPG038 (nuclear factor-κB inhibitor) and D124N in OPG099 (membrane protein CL5) in strains within the B.1.8 lineage. In addition, strains in the B.1.4 lineage showed a strong linkage between D162N in OPG040 (serpin) and R88K in OPG107 (entry-fusion complex essential component), and strains in the B.1.14 lineage exhibited a strong linkage between S288L in OPG185 (hemagglutinin) and S156L in OPG055 (protein F11). In particular, we found one linkage group in the lineage of B.1.12 composed of one synonymous substitution (V273V in OPG130) and three nonsynonymous substitutions [S532L in OPG210 (B22R family protein), D729N in OPG117 (NTPase), and G4R in OPG118 (early transcription factor 70 kDa subunit)].

We hypothesize that these observed epistatic interactions may influence the fitness of an MPXV lineage, as many of the genes with these tightly linked amino acid changes play key roles in viral infection or anti-host immunity. As a result, further functional research is warranted to elucidate the biological implications of these alterations and their epistatic effects.

### Deoptimization of codon usage in MPXV over time

Most amino acids, barring methionine and tryptophan, are encoded by at least two synonymous codons. The usage of these codons for the same amino acid is not consistent across genomes, thus resulting in codon usage bias. This bias is widely recognized for its considerable influence to the efficiency and precision of mRNA translation [35–38], mRNA stability [39], and peptide conformation [40,41], potentially impacting the adaptability and fitness of organisms. Viruses typically exhibit a lower degree of codon usage bias than their host organisms [42–44], although they generally depend on the translation machinery of host for protein synthesis. It has been hypothesized that viruses displaying weaker codon usage may demonstrate greater adaptability across diverse host species [43,45,46].

**Figure 4:**
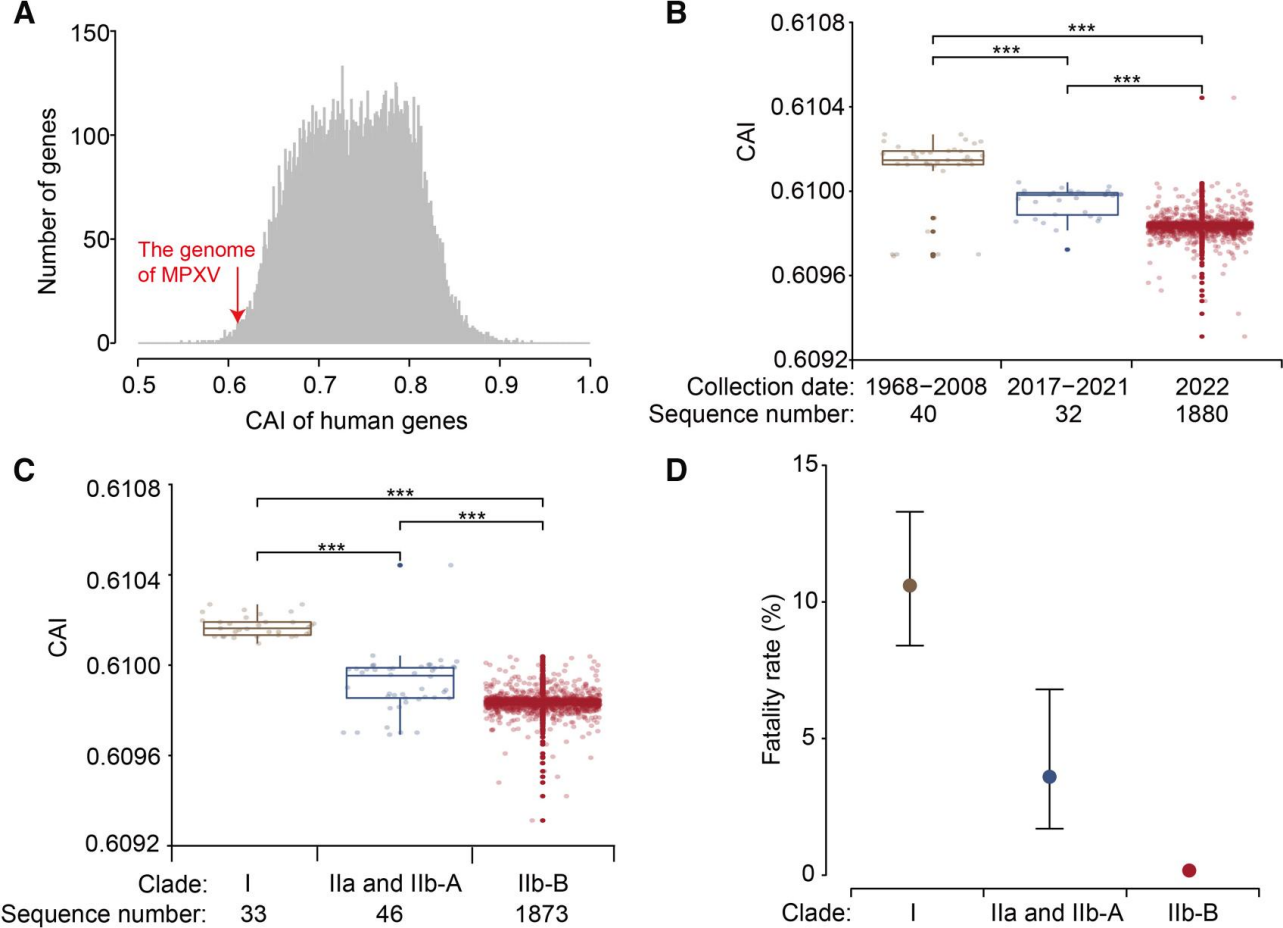
Comparisons of CAI values and fatality rates among different MPXV groups **A**. A comparative distribution of CAI values for human genes and concatenated coding sequences from the reference MPXV genome (NCBI: NC_063383). **B**. Significant differences in CAI values among three distinct MPXV variant groups, classified based on their collection dates (1968–2008, 2017–2021, and 2022). **C**. Significant differences in CAI values for MPXV variant strains belonging to different clades and lineages. **D**. Fatality rates of different MPXV clades. The fatality rates for MPXV Clade I and Clades IIa and IIb-A were obtained from a previous study [8]. The fatality rate of Clade IIb-B (0.17%) was estimated in this study. The dots on the graph represent the estimated fatality rates, while the bars indicate the 95% CIs. ***, *P* < 0.001 (Wilcoxon rank sum test); CAI, codon adaptation index; CI, confidence interval.

To examine the codon usage bias in MPXV variants, we calculated the codon adaptation index (CAI) of the concatenated coding sequences (CDSs) in each MPXV genome, following the methods described previously [44]. The CAI values for MPXV ranged from 0.6093 to 0.6104, with a median of 0.6098, and the 2.5th and 97.5th percentiles falling at 0.6098 and 0.6100, respectively. Overall, the CAI value of MPXV was substantially lower than those of human genes (**Figure 4**A), suggesting a greater tendency for MPXV to use non-preferred codons compared with human genes. This observation aligns with the understanding that MPXV genomes are A/T rich due to the general avoidance of A/T nucleotides in humans.

When we categorized the MPXV variants into three groups based on their collection dates (1968–2008, 2017–2021, and 2022), we noted a significant decrease in the CAI of MPXV over time (Figure 4B). Given that Clade I is the oldest and Clade IIb-B is the most recent, it was not surprising to find a significant difference in CAI when we split the MPXV genomes into three lineage groups: Clade I > IIa and IIb-A > IIb-B (Figure 4C). This continual deoptimization of codons in MPXV genomes was likely driven by an excess of C>T or G>A mutations, possibly as a result of APOBEC3-mediated viral editing (Table S3).

The fatality rate for MPXV Clade I was 10.6% (95% CI: 8.4%–13.3%), whereas for Clades IIa and IIb-A, it was 3.6% (95% CI: 1.7%–6.8%) [8]. Clade IIb-B, which primarily caused the 2022 MPXV outbreak, had a fatality rate of 0.17% (157 deaths out of 90,439 confirmed cases, according to the World Health Organization as of September 11th, 2023). Thus, there was a significant difference in fatality rate among the MPXV clades, with Clade I > IIa and IIb-A > IIb-B (Figure 4D). Although the decrease in fatality rate paralleled with the pattern of CAI in the three groups, it remains uncertain whether this is coincidental or indicative of a causal relationship, where the deoptimization of codon usage contributed to a decrease in fatality across the groups.

## Discussion

In this study, we explored the evolutionary patterns in protein sequences and codon usage of MPXV. Our results indicate that despite purifying selection playing a crucial role in MPXV clade differentiation, there is evidence of positive selection in the *OPG027* gene, specifically in Clade I. Moreover, we identified rapid evolution in protein sequences in the Clade IIb variants responsible for the 2022 outbreak and discovered significant epistasis among mutations within these variants. Analysis of CAI disclosed an increasing tendency of MPXV to employ less preferred human codons over time. Interestingly, a decline in fatality rates coincided with this CAI pattern. Our study offers a unique perspective on the evolutionary mechanics of MPXV.

The pathogenicity or drug resistance of viruses could be significantly influenced by only a few amino acid changes [47]. For instance, a single amino acid substitution (N752D) in the DNA polymerase of equid herpesvirus type 1 (EHV-1) significantly alters its neuropathogenicity [48]. Similarly, in the DNA polymerase (E9L) of VACV, one change (T831I) or two linked changes (A314V and A684V) substantially increase drug resistance levels [49]. Moreover, amino acid changes in the S protein of SARS-CoV-2 can significantly affect its transmission efficiency or ability to evade the immune system [32]. In this study, we found three amino acid changes (I47V, F79L, and V126I) in OPG027 (C7L), specifically in Clade I of MPXV. Since the F79 site in C7L of VACV is crucial for viral growth in human cells and engages with SAMD9 binding [29], it is likely that the F79L change, possibly in conjunction with I47V and V126I, could be linked to the unique biology of Clade I MPXV, potentially impacting its transmission, pathogenicity, or immune evasion capability. Future studies focusing on their functional aspects are necessary to confirm the biological significance of these mutations.

Similar to findings for SARS-CoV-2 [32–34], we detected many tightly linked amino acid changes in the 2022 outbreak-causing MPXV variants. These changes tend to locate in different genes, most of which are associated with viral entry or immune evasion. It is plausible that compensatory advantageous mutations occurred during the 2022 outbreak, potentially accounting for the accelerated protein sequence evolution in these MPXV variants. Yet, we cannot rule out the possibility that sampling bias or founder effects influenced the observed trends. Future research should examine the evolutionary driving mechanisms and biological significance of these epistatic interactions.

Recently, we have demonstrated that SARS-CoV-2 generally prefers less optimized human codons and produces mRNA at levels that often exceed those of genes from host cells [44]. We hypothesize that SARS-CoV-2 might employ the codon deoptimization strategy to modulate the translation rate, thereby reducing the burden on the translational machinery of host, as excessive translation activity might ultimately harm the virus itself [44]. Here, we also discovered that MPXV, similar to SARS-CoV-2, tends to utilize codons that are less preferred by human genes. To test whether MPXV genes have higher expression levels than host genes, we analyzed the RNA sequencing (RNA-seq) data from the scab of a female *Macaca fascicularis* that was infected with MPXV [Sequence Read Archive (SRA): SRR10027401] [50]. We compared the expression levels [transcripts per kilobase of exon model per million mapped reads (TPM)] of the top 5000 most abundantly expressed cellular genes to those of the MPXV genes (Figure S3). Notably, the TPM values were significantly higher for viral genes than those for cellular genes (*P* < 1E−10, Wilcoxon rank sum test), with the median TPM being 269 (95% CI: 31.5–8572) for the MPXV genes and 2.53 (95% CI: 0.67–59.9) for the cellular genes. Notably, the TPM was 345.5 for *OPG027*, which was under positive selection. These data suggest that MPXV mRNAs are more abundant than those of most host genes. Thus, it is possible that MPXV and SARS-CoV-2 use a similar strategy of codon deoptimization to control translation rates in host cells. However, we cannot exclude the alternative possibility that the reduced CAI in MPXV results from APOBEC3-mediated editing of the MPXV genomes, as C>T mutations tend to deoptimize codons in human cells [44].

We found the CAI of MPXV declined with time and differed between clades, with Clade I > IIa and IIb-A > IIb-B. It was worth noting that although the change in CAI among the three categories of MPXV strains was marginal (Figure 4C), a considerable number of mutations occurred between different clades or lineages (Figure S4) due to the high number of codons (∼ 55,000) in the MPXV genome. For example, there were ∼ 610 SNPs in the coding regions and ∼ 370 SNPs in the synonymous sites between Clades I and IIb-A, although the median CAI value was 0.6102 for Clade I and 0.6100 for Clade IIb-A (Figure S4). Therefore, the slight change in CAI of MPXV might considerably influence viral protein synthesis. The approach of attenuating viral virulence through large-scale codon deoptimization to decrease protein synthesis rates has emerged as a promising method for creating vaccine candidates, as demonstrated in the case of poliovirus and influenza A virus [51–53]. Given the urgent need for vaccines to fight against MPXV [54], this strategy could potentially be employed to develop attenuated virus-based vaccines for MPXV.

One interesting finding of this study is that the decrease in fatality rate among the three groups aligned with the CAI pattern. Given that viruses with intense translational activities could impose a significant load on host translation or trigger severe clinical symptoms [55], it is plausible that codon deoptimization in MPXV might lead to a slower virus replication rate, subsequently decreasing the fatality rate throughout evolution. Yet, we cannot disregard the likelihood that the parallel trends in CAI and fatality rates might simply be coincidental, potentially influenced by sampling bias or other confounding elements. Future investigations are needed to clarify the nature of this observed relationship.

## Materials and methods

### Evolutionary analysis

A total of 2789 MPXV genome sequences were retrieved from the NCBI [23] and GISAID (https://www.gisaid.org, as of November 13th, 2022) [24]. Only 1952 complete and high-quality genome sequences were used for downstream analysis. Mutation identification and clade assignment were performed using Nextclade v2.4.0 [25] (--input-dataset hMPXV). The mutations were annotated by SnpEff v5.0e [56] based on the reference genome (NCBI: NC_063383). Given the low assembly quality of terminal repeat regions, only the regions from 1.5 kb to 190 kb were used for phylogenetic analysis. To reduce the computing resources and time, the maximum likelihood phylogenetic tree was reconstructed by randomly sampling 370 sequences using IQ-TREE v2.2.0 (-m GTR) [57] and visualized by iTOL [58]. The divergent time of Clades IIb-A and IIb-B of MPXV was retrieved from Nextstrain (https://nextstrain.org/), and other divergent time was obtained from data reported by Babkin and his colleagues [59]. Only 756 IIb-B genomes with exact collection dates were used to estimate the mutation rates based on the phylogenetic relationships by TreeTime v0.9.4 (--reroot oldest --covariation) [31].

### Calculating pairwise divergence between MPXV strains

For each gene, we kept only one of the identical sequences and discarded sequences containing more than 15 ambiguous nucleotides or gaps. Then, we calculated *N* (the number of nonsynonymous sites), *S* (the number of synonymous sites), *dN*, *dS*, and the *dN*/*dS* (*ω*) ratio of every sequence pair by implementing the yn00 program in PAML v4 [30]. To avoid extremely large (or infinite) *ω* values resulting from the small *dS* values, only sequence pairs with *dS* values greater than 0 were under the analysis.

### Detecting signal of positive selection in *OPG027*

We analyzed 23 unique and high-quality MPXV sequences of *OPG027*, with the ortholog sequences of VARV and VACV as outgroups. We fitted the M8a (*beta* + *ω* = 1: neutral and negative selection) and M8 (*beta* + *ω* > 1: neutral, negative selection, and positive selection) models using the CODEML program in PAML v4 [30].

### The LD of Clade IIb-B

Using an in-house script, we calculated the *r*^2^ (square of the correlation coefficient) of each SNP pair outside the inverted terminal repeat regions of Clade IIb-B. Each SNP was supported by at least five genome sequences and had a frequency of less than 0.8 but more than 0.005 in Clade IIb-B. Only the SNP pairs with *r*^2^ ≥ 0.8 were selected as linked SNPs.

### Calculation of the CAI of MPXV

The CAI was calculated as previously described [44]. In brief, we weighted the frequencies of codons based on the median expression levels in 54 human tissues from the Genotype-Tissue Expression (GTEx) V8 (https://www.gtexportal.org/). Then, the CAI was calculated according to the actual frequencies of codons in the transcriptomes. We extracted the CDSs of each MPXV sequence based on the multiple sequence alignment from Nextclade [25] and the annotation of the reference genome (NCBI: NC_063383). We concatenated the CDSs of MPXV to calculate the human-expression weighted CAI value.

### Structure modelling of OPG027 (C7L)

The protein structures of OPG027 from MPXV Clades I and II were predicted by the protein structure homology modeling of SWISS-MODEL (https://swissmodel.expasy.org) [60] based on C7L protein structure of VACV from Protein Data Bank (PDB: 5CYW) [29] and visualized by PyMOL (https://pymol.org/2).

### The gene expression of MPXV

We downloaded the RNA-seq data from the scab of a female *M*. *fascicularis* infected with MPXV (SRA: SRR10027401) [50]. We calculated the relative gene expression of MPXV and its host by Kallisto v0.44.0 [61], using the longest CDSs of *M*. *fascicularis* (Macaca_fascicularis_6.0) genes and the CDSs of MPXV genes (NCBI: NC_063383) as the reference sequences.

## Supplementary Material

qzad003_Supplementary_Data

## CRediT author statement

**Ke-Jia Shan**: Formal analysis, Investigation, Data curation, Visualization, Writing – original draft, Writing – review & editing. **Changcheng Wu**: Formal analysis, Investigation, Data curation, Visualization, Writing – original draft, Writing – review & editing. **Xiaolu Tang**: Investigation, Writing – original draft, Writing – review & editing. **Roujian Lu**: Writing – review & editing. **Yaling Hu**: Resources, Writing – review & editing. **Wenjie Tan**: Conceptualization, Resources, Supervision, Writing – original draft, Writing – review & editing. **Jian Lu**: Conceptualization, Resources, Supervision, Writing – original draft, Writing – review & editing. All authors have read and approved the final manuscript.
