# Supplementary material for: Molecular Evolution of Protein Sequences and Codon Usage in Monkeypox Viruses
Source: Genomics Proteomics Bioinformatics. 2023 Dec 12;22(1):qzad003. doi: 10.1093/gpbjnl/qzad003 (PMC11425058; doi:10.1093/gpbjnl/qzad003)
Supplement: qzad003_Supplementary_Data [file qzad003_supplementary_data.zip › Supplementary captions.docx]

**Supplementary material**

**Figure S1 Predicted protein structures of OPG027 from MPXV based on C7L from vaccinia virus**

**A.** Amino acid sequence alignment of OPG027 from MPXV (Clade I and II) and C7L from vaccinia virus. The predicted protein structures of OPG027 from MPXV Clade I (**B**) and Clade II (**C**), with highlighted amino acid changes between Clade I and II.

**Figure S2** **Linkage disequilibrium between SNPs in Clade IIb-B of MPXV**

The *r^2^* (Y-axis) against the distance between each SNP pair (X-axis). The SNP pairs in the 15 linked SNP groups (*r^2^* ≥ 0.8) included in Figure 3 are shown in red dots. SNPs, single nucleotide polymorphisms.

**Figure S3 The distribution of gene expression levels (Log_10_-transformed TPM) for host genes and MPXV genes**

The RNA-seq data was obtained from the scab of a female *Macaca fascicularis* infected with MPXV (Sequence Read Archive accession number: SRR10027401). The expression level of *OPG027*, which was under positive selection, is also indicated. TPM, transcripts per kilobase of exon model per million mapped reads; RNA-seq, RNA sequencing.

**Figure S4**  **The number of SNPs in the coding regions in different clades of MPXV**

The total number of SNPs in the coding regions (**A**) and synonymous sites (**B**) in a clade of MPXV, using the NC_063383 (Clade IIb-A) as the reference genome. ****, *P* < 0.0001 (Wilcoxon rank sum test).

**Table S1 The number of sequences with mutations in *OPG027* among different clades of MPXV**

**Table S2 The linkage group of mutations in Clade IIb-B of MPXV**

**Table S3 Summary of SNPs in Clade IIb-B of MPXV**

**Table S4 The MPXV genome sequences downloaded from the NCBI**

**Table S5 The MPXV genome sequence downloaded from GISAID**
