# Supplementary material for: Molecular Evolution of Protein Sequences and Codon Usage in Monkeypox Viruses
Source: Genomics Proteomics Bioinformatics. 2023 Dec 12;22(1):qzad003. doi: 10.1093/gpbjnl/qzad003 (PMC11425058; doi:10.1093/gpbjnl/qzad003)
Supplement: qzad003_Supplementary_Data [file qzad003_supplementary_data.zip › Table S1-done.docx]

**Table S1 The number of sequences with mutations in *OPG027* among different**

**clades of MPXV**

| **SNP in the genome** | **SNP in the gene coding strand** | **Type** | **Amino acid**  **change** | **Clade** | | | |
| --- | --- | --- | --- | --- | --- | --- | --- |
|  |  |  |  | **I** | **IIa** | **IIb-A** | **IIb-B** |
| G16333A | C45T | Syn | I15I | - | - | - | 1/1873 |
| C16327T | G51A | Syn | L17L | - | - | - | 2/1873 |
| T16239C | A139G | Nonsyn | I47V | 33/33 | - | - | - |
| C16227T | G151A | Nonsyn | D51N | - | - | 1/32 | - |
| G16213A | C165T | Syn | I55I | - | - | - | 1/1873 |
| C16209T | G169A | Nonsyn | E57K | - | - | - | 1/1873 |
| G16147A | C231T | Syn | Y77Y | 1/33 | - | - | - |
| G16141T | C237A | Nonsyn | F79L | 32/33 | - | - | - |
| C16125T | G253A | Nonsyn | E85K | - | - | - | 1/1873 |
| C16123A | G255T | Nonsyn | E85D | 12/33 | - | - | - |
| G16110A | C268T | Syn | L90L | - | - | - | 11/1873 |
| G16102A | C276T | Syn | N92N | 33/33 | - | - | - |
| C16002T | G376A | Nonsyn | V126I | 33/33 | - | - | - |
| C15947T | G431A | Nonsyn | R144K | - | - | - | 1/1873 |

*Note*: Within each lineage, the fraction is represented as the number of genome sequences containing the mutation over the total number of genome sequences. The SNPs were obtained based on the MPXV reference genome (NCBI: NC_063383). Note that *OPG027* is positioned on the antisense strand of the MPXV genome. The first column corresponds to the sense strand of the MPXV genome, while the second column corresponds to the coding strand of *OPG027*. Syn, synonymous; Nonsyn, nonsynonymous; SNP, single nucleotide polymorphism.
