# Supplementary material for: Molecular Evolution of Protein Sequences and Codon Usage in Monkeypox Viruses
Source: Genomics Proteomics Bioinformatics. 2023 Dec 12;22(1):qzad003. doi: 10.1093/gpbjnl/qzad003 (PMC11425058; doi:10.1093/gpbjnl/qzad003)
Supplement: qzad003_Supplementary_Data [file qzad003_supplementary_data.zip › Table S2-done.docx]

**Table S2 The linkage group of mutations in Clade IIb-B of MPXV**

| **Linkage group (Lineage)** | **Position** | **SNP** | **Amino acid change** | **Type** | **Gene** | **Protein** |
| --- | --- | --- | --- | --- | --- | --- |
| Group 1  (B.1.11) | 67611 | G>A | Y307Y | Syn | *OPG085* | Metalloendopeptidase |
|  | 130231 | G>A | Q436Q | Syn | *OPG151* | DNA-dependent RNA polymerase subunit rpo132 |
|  | 18133 | C>T | P232P | Syn | *OPG031* | C4L/C10L-like family protein |
|  | 159277 | G>A | E121K | Nonsyn | *OPG185* | Hemagglutinin |
| Group 2  (B.1) | 103417 | C>T | E552K | Nonsyn | *OPG123* | Nucleoside triphosphatase I |
|  | 173114 | C>T |  | Intergenic | *OPG200*-*OPG204* | |
| Group 3  (B.1.13) | 132520 | C>T |  | Intergenic | *OPG151*-*OPG153* | |
|  | 175093 | G>A | D188N | Nonsyn | *OPG204* | Interferon-alpha/beta-receptor-like secreted glycoprotein |
| Group 4  (B.1.3) | 64426 | C>T | T97T | Syn | *OPG083* | Viral core cysteine proteinase |
|  | 55133 | G>A | R665C | Nonsyn | *OPG074* | Intracellular enveloped virion morphogenesis protein |
| Group 5  (B.1.8) | 63811 | G>A | F302F | Syn | *OPG083* | Viral core cysteine proteinase |
|  | 22643 | G>A | H173Y | Nonsyn | *OPG038* | Nuclear factor-κB inhibitor |
|  | 80111 | C>T | T37T | Syn | *OPG103* | DNA-directed RNA polymerase subunit |
|  | 78034 | G>A | D124N | Nonsyn | *OPG099* | Membrane protein CL5 |
| Group 6  (B.1) | 156429 | C>T |  | Intergenic | *OPG181*-*OPG185* | |
|  | 65571 | C>T | L284L | Syn | *OPG084* | RNA helicase NPH-II (2) |
|  | 144988 | G>A | L140L | Syn | *OPG167* | Cluster of differentiation 47-like protein |
| Group 7  (B.1.4) | 189258 | G>A | L20L | Syn | *OPG005* | B-cell lymphoma 2-like protein |
|  | 148427 | G>A | F323F | Syn | *OPG174* | Hydroxysteroid dehydrogenase |
|  | 24995 | C>T | D162N | Nonsyn | *OPG040* | Serpin |
|  | 85774 | G>A | R88K | Nonsyn | *OPG107* | Entry-fusion complex essential component |
| Group 8  (B.1) | 116130 | G>A | K127K | Syn | *OPG134* | Intermediate transcription factor VITF-3 (1) |
|  | 152202 | G>A |  | Intergenic | *OPG178*-*OPG180* | |
| Group 9  (B.1.14) | 159779 | C>T | S288L | Nonsyn | *OPG185* | Hemagglutinin |
|  | 36617 | G>A | S156L | Nonsyn | *OPG055* | Protein F11 |
| Group 10  (B.1.12) | 182950 | C>T | S532L | Nonsyn | *OPG210* | B22R family protein |
|  | 111084 | G>A | V273V | Syn | *OPG130* | A5L protein-like |
|  | 98233 | G>A | D729N | Nonsyn | *OPG117* | NTPase (1) |
|  | 98455 | G>A | G4R | Nonsyn | *OPG118* | Early transcription factor 70 kDa subunit |
| Group 11  (B.1) | 51378 | G>A | L263L | Syn | *OPG070* | Membrane protein E8 |
|  | 181082 | G>A | D189N | Nonsyn | *OPG209* | Virulence protein |
|  | 89030 | C>T | V140I | Nonsyn | *OPG109* | RNA polymerase-associated transcription-specificity factor RAP94 |
|  | 16110 | G>A | L90L | Syn | *OPG027* | Host range protein; Type I interferon inhibitor |
| Group 12  (B.1) | 169928 | G>A | D87N | Nonsyn | *OPG197* | CPXV205 protein |
|  | 58518 | G>A | F22F | Syn | *OPG077* | Telomere-binding protein I1 |
| Group 13  (B.1.10) | 89906 | C>T | S92F | Nonsyn | *OPG110* | Late transcription factor VLTF-4 (1) |
|  | 94798 | G>A | E47K | Nonsyn | *OPG115* | Virion core protein D3 |
| Group 14  (B.1.9, B.1.1, B.1) | 82405 | CAA>CCT | Q427P | Nonsyn | *OPG105* | DNA-dependent RNA polymerase subunit rpo147 |
|  | 82406 |  |  |  |  |  |
| Group 15  (B.1.1) | 119838 | C>T | I79I | Syn | *OPG137* | Viral membrane formation protein |
|  | 52232 | G>A | S736L | Nonsyn | *OPG071* | DNA polymerase (2) |
