# Supplementary material for: Molecular Evolution of Protein Sequences and Codon Usage in Monkeypox Viruses
Source: Genomics Proteomics Bioinformatics. 2023 Dec 12;22(1):qzad003. doi: 10.1093/gpbjnl/qzad003 (PMC11425058; doi:10.1093/gpbjnl/qzad003)
Supplement: qzad003_Supplementary_Data [file qzad003_supplementary_data.zip › Table S3-done.docx]

**Table S3 Summary of SNPs in Clade IIb-B of MPXV**

| **Type of SNPs** | **Synonymous SNPs** | **Nonsynonymous SNPs** |
| --- | --- | --- |
| C>T | 244 (43.7%) | 454 (43.2%) |
| G>A | 252 (45.2%) | 387 (36.9%) |
| Other types | 62 (11.1%) | 209 (19.9%) |
| Total number of SNPs | 558 | 1050 |
