# Supplementary figures and images for: Molecular Evolution of Protein Sequences and Codon Usage in Monkeypox Viruses
Source: Genomics Proteomics Bioinformatics. 2023 Dec 12;22(1):qzad003. doi: 10.1093/gpbjnl/qzad003 (PMC11425058; doi:10.1093/gpbjnl/qzad003)

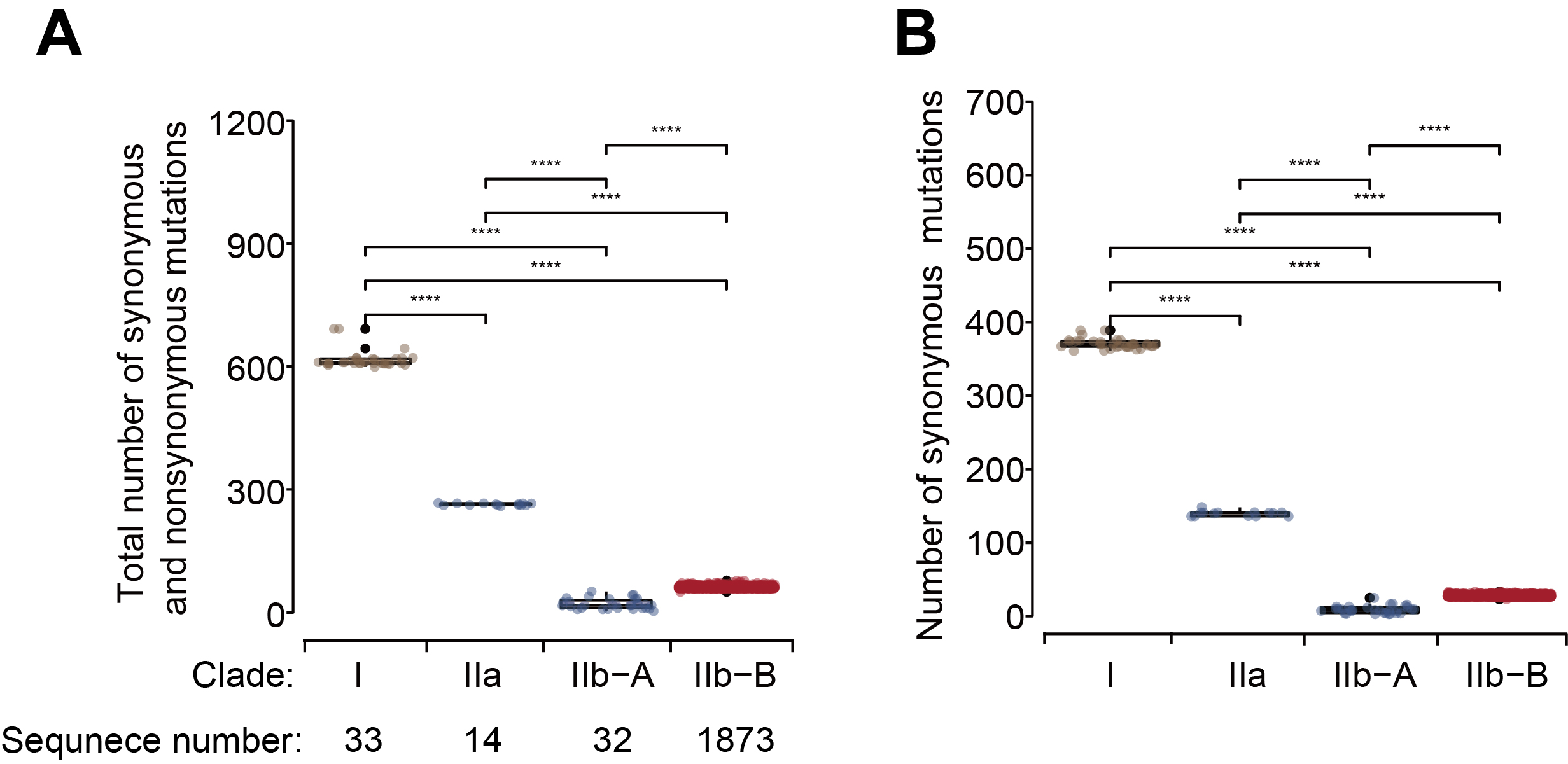

Supplement: qzad003_Supplementary_Data [file qzad003_supplementary_data.zip › FigureS4.jpg]

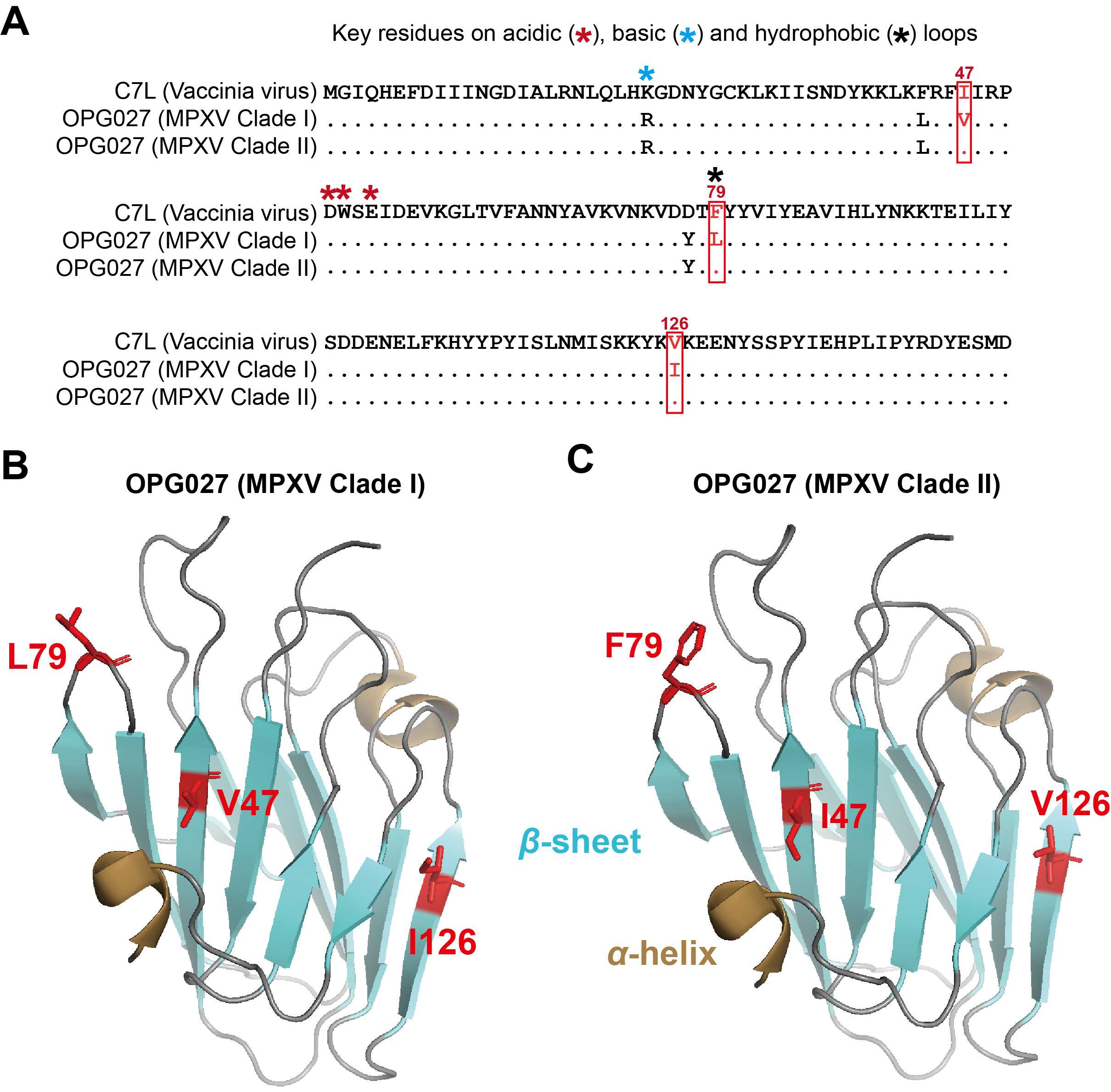

Supplement: qzad003_Supplementary_Data [file qzad003_supplementary_data.zip › FigureS1.jpg]

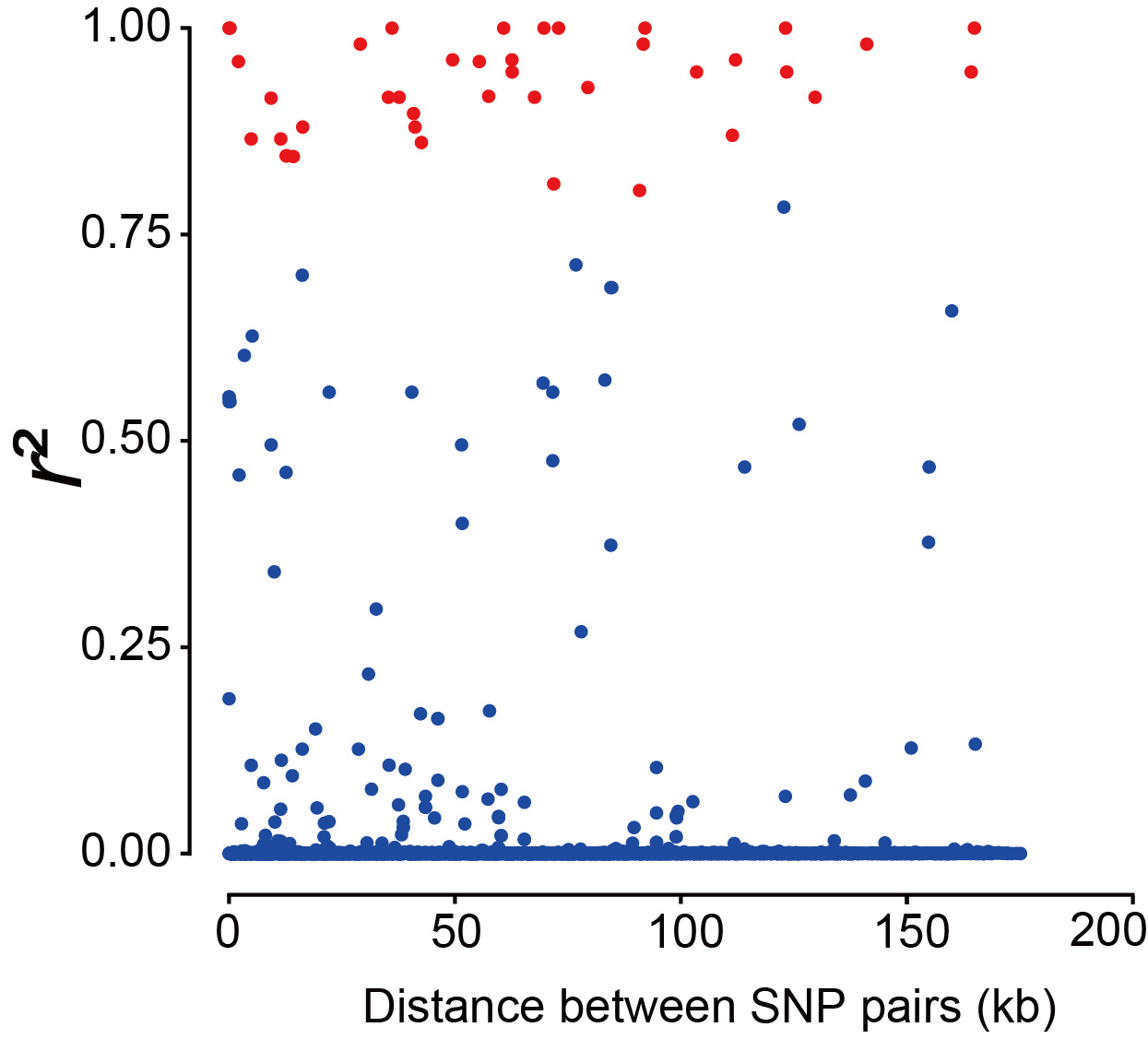

Supplement: qzad003_Supplementary_Data [file qzad003_supplementary_data.zip › FigureS2.jpg]

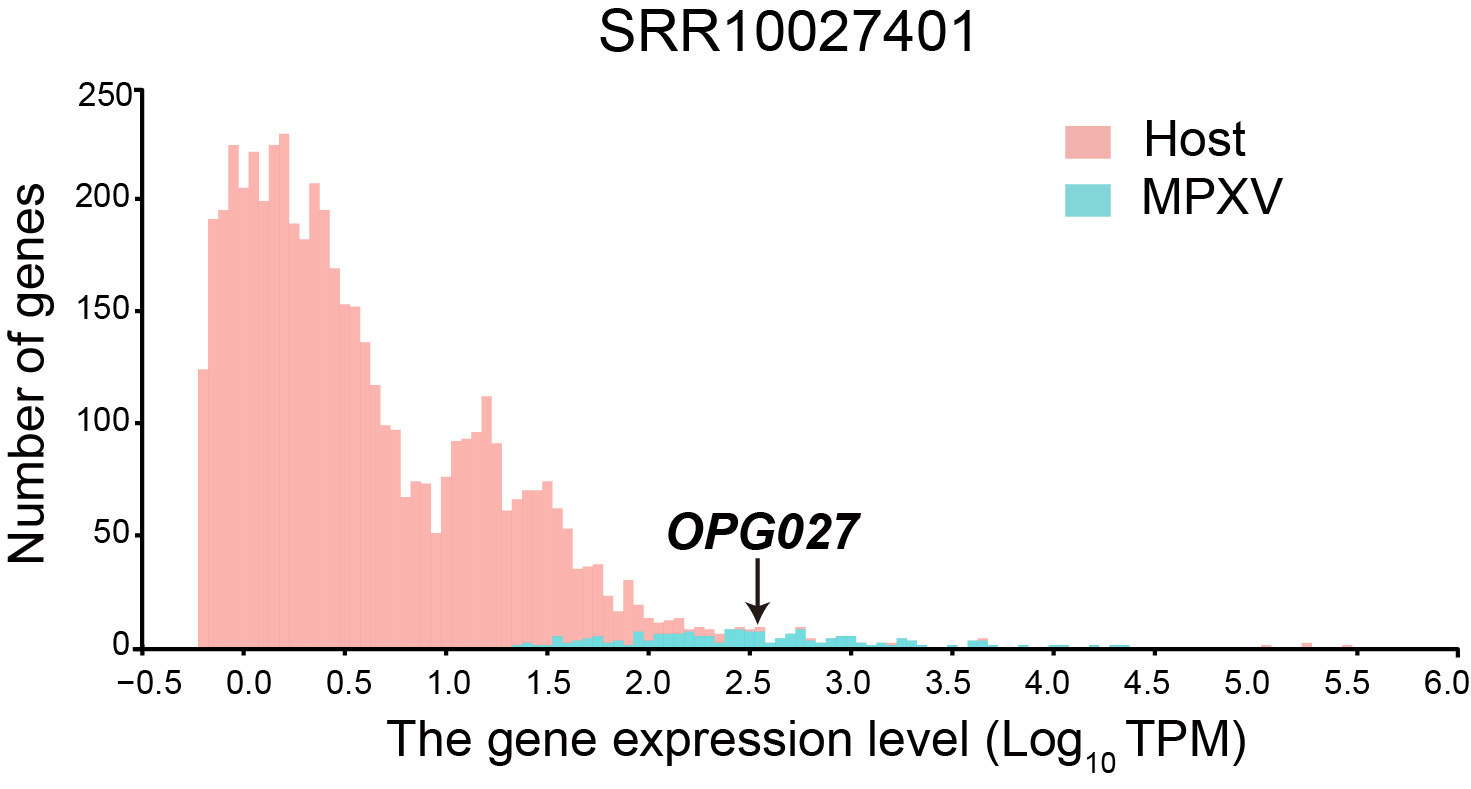

Supplement: qzad003_Supplementary_Data [file qzad003_supplementary_data.zip › FigureS3.jpg]
